# Cadaveric Simulation of Otologic Procedures: An Analysis of Droplet Splatter Patterns During the COVID-19 Pandemic

**DOI:** 10.1177/0194599820930245

**Published:** 2020-05-19

**Authors:** Dhruv Sharma, Kolin E. Rubel, Michael J. Ye, Vincent J. Campiti, Aaron E. Carroll, Jonathan Y. Ting, Elisa A. Illing, Sarah J. Burgin

**Affiliations:** 1 Department of Otolaryngology–Head and Neck Surgery, Indiana University, Indianapolis, Indiana, USA; 2 Department of Pediatrics, Indiana University, Indianapolis, Indiana, USA

**Keywords:** COVID-19, droplets, splatter, myringotomy, mastoidectomy, otologic surgery

## Abstract

**Objective:**

The otolaryngology community has significant concerns regarding the spread of SARS-CoV-2 through droplet contamination and viral aerosolization during head and neck examinations and procedures. The objective of this study was to investigate the droplet and splatter contamination from common otologic procedures.

**Study Design:**

Cadaver simulation series.

**Setting:**

Dedicated surgical laboratory.

**Methods:**

Two cadaver heads were prepped via bilateral middle cranial fossa approaches to the tegmen (n = 4). Fluorescein was instilled through a 4-mm burr hole drilled into the middle cranial fossa floor, and presence in the middle ear was confirmed via microscopic ear examination. Myringotomy with ventilation tube placement and mastoidectomy were performed, and the distribution and distance of resulting droplet splatter patterns were systematically evaluated.

**Results:**

There were no fluorescein droplets or splatter contamination observed in the measured surgical field in any direction after myringotomy and insertion of ventilation tube. Gross contamination from the surgical site to 6 ft was noted after complete mastoidectomy, though, when performed in standard fashion.

**Conclusion:**

Our results show that there is no droplet generation during myringotomy with ventilation tube placement in an operating room setting. Mastoidectomy, however, showed gross contamination 3 to 6 ft away in all directions measured. Additionally, there was significantly more droplet and splatter generation to the left of the surgeon when measured at 1 and 3 ft as compared with all other measured directions.

The current global pandemic brought about by the novel coronavirus disease 2019 (COVID-19) has led to sweeping transformative change in the health care sector. US hospitals have essentially ceased all elective, nonurgent surgical cases in accordance with guidelines from the Centers for Disease Control and Prevention,^1^ and much uncertainty remains on how to resume safely. In the current climate, the safety of otolaryngology procedures is of particular concern, as current evidence suggests elevated risk due to close contact with upper respiratory mucosa, which harbors a high viral load.^2-4^

Viral transmission is thought to be primarily via respiratory droplets,^5^ which can travel >2 m and linger on contaminated surfaces for hours, if not days.^6^ This has led to significant concern for the transmission of the novel coronavirus due to aerosol-generating procedures.^7^ As a result, the American Academy of Otolaryngology–Head and Neck Surgery has issued a position statement to limit elective procedures requiring interaction with upper airway mucosal surfaces or those with increased risk of aerosolization, which may include otologic procedures such as myringotomy and mastoidectomy.^8,9^

However, to our knowledge, no published literature exists to guide decision making on the safety of these common otologic procedures. This is an important area of investigation due to the potential for the middle ear and mastoid to harbor respiratory pathogens^10^ and for droplet dispersion and aerosol generation with use of high-speed drills.^11^ This study seeks to investigate and clarify these risks by evaluating droplet dispersion patterns resulting from otologic procedures in a cadaver-simulated series.

## Materials and Methods

### Supplies and Equipment

The study was exempt from institutional review board because it involved the use of nonliving human cadaveric tissue specimens (IRB protocol 2004100753). The experiments in this study were all conducted in a dedicated surgical laboratory on 2 fresh-frozen cadaver head specimens prepared in identical fashion and placed in a standard position for the procedures.

With the following technique, a middle cranial fossa (MCF) approach was performed bilaterally on both specimens to expose the floor of the MCF. A posteriorly based trapdoor incision approximately 6 × 8 cm was made superior to the auricle down to the calvarium, and then a 6 × 6–cm bone flap, centered above the temporal root of the zygoma, was fashioned with a 4-mm cutting burr. After the MCF floor was completely exposed, a 4-mm port was drilled into the middle ear through the tegmen.

**Figure 1.**
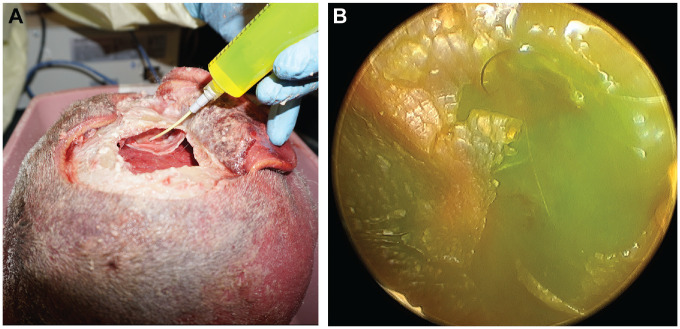
(A) Instillation of fluorescein into the middle ear via the middle cranial fossa approach. (B) Endoscopic confirmation of fluorescein behind the tympanic membrane.

Fluorescein solution at a concentration of 1 mg/mL was created by mixing 500 mg of fluorescein (10% [100 mg/mL], fluorescein injection, USP; AK-Fluor) with 495 mL of sterile saline. The 1 mg/mL fluorescein solution was instilled with a 14-gauge angiocath through the port into the middle ear space (**Figure 1A**). The presence of fluorescein in the middle ear space was confirmed endoscopically by visualization through the external auditory canal (**Figure 1B**).

### Experimental Setup

**Figure 2.**
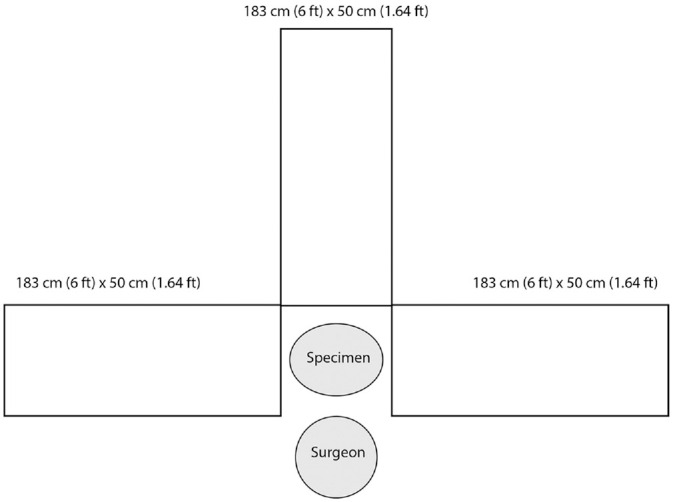
Schematic of experimental setup for cadaveric simulation.

Each cadaver head was placed in the standard otologic position. Procedures were performed by right-handed surgeons (D.S., M.J.Y.). Three sets of nonabsorbent blue paper (183 cm [6 ft] × 50 cm [1.64 ft]) affixed to a rigid backing were placed 90° from each other in the following directions: (1) left of the surgeon, (2) anterior to the head or across from the surgeon, and (3) right of the surgeon (**Figure 2**). A 25 × 25–cm piece of nonabsorbent blue paper was also affixed to the surgeon’s gown on the chest. The surgeon additionally wore a face shield throughout the procedure.

### Experiment

The following surgical procedures were performed systematically on each head: (1) left-sided myringotomy with insertion of a ventilation tube; (2) left-sided complete mastoidectomy, including entry into the mastoid antrum and exposure of the tegmen, sigmoid sinus, and lateral semicircular canal; (3) right-sided myringotomy with insertion of a ventilation tube; and (4) right-sided complete mastoidectomy. A Stryker S2 πDrive Drill with a 6-mm Multi Flute burr was utilized for each mastoidectomy procedure. **Table 1** summarizes the procedures that were performed on the 2 cadaver heads and the duration.

**Table 1.** Droplet Splatter Results. ^a^

Procedure	Duration	Droplet or splatter contamination	Across	Left	Right	Chest
Left MVT						
Cadaver 1	57 s	No	0	0	0	0
Cadaver 2	51 s	No	0	0	0	0
Right MVT						
Cadaver 1	55 s	No	0	0	0	0
Cadaver 2	48 s	No	0	0	0	0
Left mastoidectomy						
Cadaver 1	31 m, 9 s	Yes				108
1 ft			36	625	190	
3 ft			11	51	4	
6 ft			1	1	0	
Cadaver 2	7 m, 0 s	Yes				2
1 ft			160	625	176	
3 ft			2	56	12	
6 ft			2	5	0	
Right mastoidectomy						
Cadaver 1	9 m, 25 s	Yes				188
1 ft			34	577	236	
3 ft			4	47	11	
6 ft			0	0	4	
Cadaver 2	5 m, 26 s	Yes				3
1 ft			115	599	201	
3 ft			6	17	13	
6 ft			0	0	9	

Abbreviation: MVT, myringotomy with ventilation tube placement.

a Directions specified with respect to the operating surgeon.

Following each surgical procedure, the number and distance of the droplets and splatter on the nonabsorbent blue paper was evaluated and measured by the following technique. Transparent grid graphs (25 × 25 cm) were laid side-by-side at 1, 3, and 6 ft from the surgical site. The blue paper on the surgeon’s chest was removed and laid flat, and a grid was placed on it as well. The surgeon’s face shield was removed and laid flat, and blue paper with an overlying grid was placed underneath it.

Since fluorescein fluoresces yellow under ultraviolet light and blue paper does not, the evaluators used an ultraviolet light to visualize the droplets and splatter from each experimental condition. The evaluators then counted and recorded the number and distance of any 1-cm^2^ area containing any illuminated fluorescent spot or any gross contamination. Fluorescein did not penetrate the bone but was limited to the mucosa.

## Results

No observable fluorescein droplets were noted in the measured surgical field in any direction after myringotomy and insertion of ventilation tube. Visible fluorescein contamination was noted only on surfaces in direct contact with surgical instruments. In contrast, gross contamination was measured 3 ft in all cardinal directions after every mastoidectomy. The number of droplets identified at 1 and 3 ft to the left of the surgeon was significantly greater than the number on the right of the surgeon or across from the surgeon. The right side of the surgeon had significantly more splatter and droplets than across at 1 ft (**Table 2**). After each mastoidectomy, the surgical field within 6 in, the hands and arms, the face shield, and the chest were grossly contaminated by droplets and splatter.

**Table 2.** Droplet Splatter Analysis.

				Two-tailed *t* test		
Distance, ft	Across	Left	Right	Left vs right	Left vs across	Right vs across
1	86.25	606.50	200.75	<.0001^a^	< .0001^a^	.0142^b^
3	5.75	42.75	10.00	.0109^b^	.0062^a^	.1812
6	0.75	1.50	3.25	.5010	.5801	.2969

a *P* < .01.

b *P* < .05.

## Discussion

COVID-19 has rapidly disseminated from the Hubei province of China across the globe, with over 3 million confirmed cases in 212 countries as of April 29, 2020.^12^ The primary mode of viral transmission of SARS-CoV-2 is believed to be through the spread of respiratory droplets, which has led to significant community spread of the disease.^5^ The potential for spread through opportunistic aerosolization during aerosol generation procedures is also a concern. Since the upper respiratory tract harbors a high viral load,^3^ otolaryngologists are vulnerable to SARS-CoV-2 transmission while performing head and neck procedures that utilize suction and powered instrumentation, such as the surgical drill, especially if they are doing so without appropriate protective personal equipment.^4^ With its connection to the nasopharynx through the eustachian tube, the middle ear can serve as a possible source of transmission for upper respiratory tract pathogens^10^ during routine otologic procedures, such as myringotomy and mastoidectomy. With the persistence of SARS-CoV-2 in the general population for the foreseeable future, we will need to navigate these risks as we resume elective surgical procedures and perform urgent operations on patients whose SARS-CoV-2 status is unknown or positive.

In conducting this cadaveric simulation study, we confirmed that performing a myringotomy with insertion of ventilation tube caused no droplet or splatter contamination. The potential for aerosolization remains, however, when suction is used across a mucosal surface. In contrast, a complete mastoidectomy performed in standard fashion resulted in gross contamination up to 6 ft from the surgical site, which was the farthest distance measured. Aerosol generation with surgical drills has been established in the orthopedic literature.^13^ This is likely secondary to the nature of the operation, which involves high-speed drilling of the temporal bone under irrigation creating visible splatter from bone dust and irrigation droplets. Our study also demonstrated that significantly more droplet and splatter occur to the left of the surgeon, which corresponds to the direction of rotation of the drill. Those within 1 to 3 ft of the drill are at increased risk of exposure. In teaching institutions where multiple members of the team may be directly adjacent to the primary surgeon, this must be taken into account. While the drill is being operated, all steps should be taken to reduce the number of other people within a 3- to 6-ft radius.

Several limitations to this cadaveric simulation study deserve consideration. These procedures were not conducted in a normal adult clinic setting with an actively respiring patient. With stimulation of the external auditory canal during examination, patients can produce a cough reflex that may cause increased risk of viral transmission. Moreover, there was no assessment of aerosolization, either forced (eg, sneezing) or from drilling, in this experimental model. However, we believe that it is still vital to understand the quantity, quality, and range of droplet and splatter contamination involved during these common procedures, as respiratory droplets are considered to be the primary mode of SARS-CoV-2 transmission. Another limitation is that only droplets and splatter visible to the human eye were measured. Furthermore, instead of a complete 360° assessment, the design model allowed for measurements only in the cardinal directions surrounding the specimen.

In the context of the findings from this study, we believe that it is important to devise techniques to limit the spread of gross contamination from mastoid surgery. This will not be easily accomplished, because it is difficult to operate a microscope while wearing a face shield or powered air-purifying respiratory. Risks to the rest of the surgical staff and anesthesia team also are present in the operating room, which highlights that additional protective personal equipment is necessary for the surgical team, not just the operating surgeon. Carron et al recently published a simple technique involving the use of 2 readily available clear surgical drapes to control droplet and splatter contamination during mastoidectomy.^14^ They reported that surgical visualization was not affected. Although a good step in the right direction for preventing the spread of gross contaminant, this methodology does not create an air-tight surgical field, and droplet or splatter contamination was not measured in any objective way. Further studies should be performed comparing different techniques to determine the extent of prevention of droplet contamination and aerosolization.

## Conclusion

It is essential to evaluate all procedures that have a risk of disrupting respiratory epithelium and spreading SARS-CoV-2. Our results indicate that there is no droplet generation during myringotomy with ventilation tube placement in an operating room setting. For mastoidectomy, however, gross contamination was visualized 3 to 6 ft away in all cardinal directions, and significantly more occurred on the left side of the surgeon when compared with the other sides, corresponding to the direction of drill rotation. It is critical to develop techniques to contain contamination as much as possible.
